# A Low Mismatch Current Charge Pump Applied to Phase-Locked Loops

**DOI:** 10.3390/mi15070913

**Published:** 2024-07-14

**Authors:** Min Guo, Lixin Wang, Shixin Wang, Jiacheng Lu, Mengyao Cui

**Affiliations:** 1 Institute of Microelectronics of the Chinese Academy of Sciences, Beijing 100029, China; guomin@ime.ac.cn (M.G.); wangshixin2020@ime.ac.cn (S.W.); lujiacheng@ime.ac.cn (J.L.); cuimengyao@ime.ac.cn (M.C.); 2 University of Chinese Academy of Sciences, Beijing 100049, China

**Keywords:** integrated circuits (ICs), charge pump, current mismatch, phase-locked loop (PLL), charge sharing

## Abstract

This paper presents a charge pump circuit with a wide output range and low current mismatch applied to phase-locked loops. In this designed structure, T-shaped analog switches are adopted to suppress the non-ideal effects of clock feedthrough, switching time mismatch, and charge injection. A source follower and current splitting circuits are proposed to improve the matching accuracy of the charging and discharging currents and reduce the current mismatch rate. A rail-to-rail high-gain amplifier with a negative feedback connection is introduced to suppress the charge-sharing effect of the charge pump. A cascode current mirror with a high output impedance is used to provide the charge and discharge currents for the charge pump, which not only improves the current accuracy of the charge pump but also increases the output voltage range. The proposed charge pump is designed and simulated based on a 65 nm CMOS process. The results show that when the power supply voltage is 1.2 V, the output current of the charge pump is 100 μA, the output voltage is in the range of 0.2~1 V, and the maximum current mismatch rate and current variation rate are only 0.21% and 1.4%, respectively.

## 1. Introduction

A phase-locked loop (PLL) is an electronic system that utilizes feedback control mechanisms to synchronize the frequency and phase. It plays a pivotal role in modern communication and electronic engineering. PLLs are used in a variety of circuits, including frequency synthesizers, clock skew correction, clock data recovery, and jitter filtering applications. PLLs improve the system performance and stability by precisely controlling signals to maintain their frequency and phase consistency with the reference signal [1]. In a System on a Chip (SoC), Analog-to-Digital Converters (ADCs) and Digital-to-Analog Converters (DACs) serve as the links between the digital and analog worlds, while Phase-Locked Loop (PLL) frequency synthesizers provide a system’s clock signals [2,3]. Among these, the charge pump phase-locked loop (CP-PLL) has various advantages, including a wide capture range, a small phase variance during locking, and low power consumption, enabling it to achieve zero static phase errors in theory. It has become one of the most widely used architectures in various high-speed systems, including microprocessors and communication networks [4,5,6,7].

**Figure 1:**
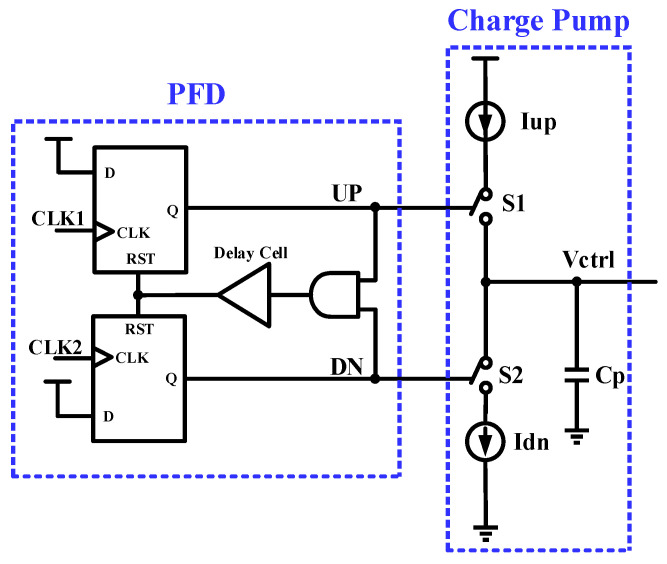
PFD with charge pump.

The charge pump is the CP-PLL’s central component, and its major job is to transform the digital pulse produced by the phase-frequency detector (PFD) into a steady analog voltage. This analog voltage is then utilized to modify the output frequency of the voltage-controlled oscillator (VCO), resulting in frequency locking throughout the CP-PLL loop. With this approach, the charge pump allows the phase-locked loop to convert a digital signal into an analog control quantity, accurately regulating the frequency of the VCO and guaranteeing the system reaches the correct synchronization [8]. As shown in Figure 1, a charge pump can be viewed as a circuit consisting of two current sources and two sets of switches. The current sources are the charging current Iup and the discharging current Idn, and their current values are the same. The switches S1 and S2 are controlled to be turned on and off by the output UP and DN pulse signals of the phase and frequency detector (PFD). The purity of the control voltage Vctrl in the filter capacitor Cp impacts the quality of the VCO output signal, which, in turn, impacts the system’s overall performance. Therefore, it is essential to build a charge pump with exceptional stability and accuracy.

Reference spurs are mostly caused by non-ideal effects of the charge pump circuit, such as current mismatch, switching time mismatch, charge sharing, and clock feedthrough. These non-ideal effects will have varied degrees of impact on the operation of the charge pump circuit. A phase-locked loop circuit will experience periodic jitter due to these non-ideal effects, which will lower the purity of the charge pump output voltage and produce noise at the VCO output [9,10,11]. The total phase offset due to the current mismatch can be approximated by [12].

(1) $$\begin{array}{l} \Delta \phi_{\mathrm{tol}}=2\pi (\Delta \varphi_{\mathrm{leak}}+\Delta \varphi_{\mathrm{mismatch}}+\Delta \varphi_{\mathrm{timing}})\\ =2\pi (\frac{I_{\mathrm{leak}}}{I_{\mathrm{cp}}}+\frac{\Delta I}{I_{\mathrm{cp}}}\cdot \frac{T_{\mathrm{switch}}}{T_{\mathrm{ref}}}+\frac{\Delta T\cdot T_{\mathrm{switch}}}{{T_{\mathrm{ref}}}^{2}}) \end{array}$$

where *I*_cp_ is the charge pump current, *I*_leak_ is the leakage current, *T*_ref_ is the reference clock period, *T*_switch_ is the time at which the charge pump is turned off in the lock state, and ∆*I* and ∆*T* are the current and timing mismatches, respectively. Leakage current can be ignored under a large *I*_cp_ value; therefore, it is crucial to minimize the current mismatch in the CP design.

Several techniques for minimizing the non-ideal effects of the charge pump have been proposed in the literature [11,13,14,15]. To improve the charge pump performance and reduce the impact of non-ideal effects, reference [13] proposed a novel charge pump based on a constant transconductance rail-to-rail operational amplifier, which significantly reduces the adverse effects of current mismatch and charge sharing on the performance of the charge pump. The maximum current mismatch was reduced from 26.3% to 5.4% compared to that of a conventional charge pump. In reference [14], a new charge pump design using a feedback loop to reduce random mismatch was proposed. Koithyar et al. [15] proposed a new Integer-N charge pump phase-locked loop in 2020, whose charge pump uses two high-gain amplifiers and a transmission gate structure to improve the clock feedthrough and current mismatch effects. Compared with traditional structures, the current mismatch of this charge pump was reduced by 3.21%. Abdul et al. [11] proposed a charge pump structure with two compensators, using linearization techniques to control and compensate for the mismatch between currents (charging and discharging).

In this work, a charge pump circuit with a wide output range and low current mismatch applied to phase-locked loops is proposed. T-shaped analog switches are used to minimize non-ideal effects. Two sets of source followers and current splitting circuits are incorporated to improve the matching accuracy for the charge and discharge currents and reduce current mismatch. The Section 2 describes the conventional charge pump structure and analyzes the impact of non-ideal effects. The Section 3 describes the proposed charge pump circuit in detail. The Section 4 analyzes the simulation results. The Section 5 is a summary of the work.

## 2. Conventional Charge Pump Circuits and Non-Ideality Analysis

### 2.1. A Conventional Op-Amp Charge Pump

**Figure 2:**
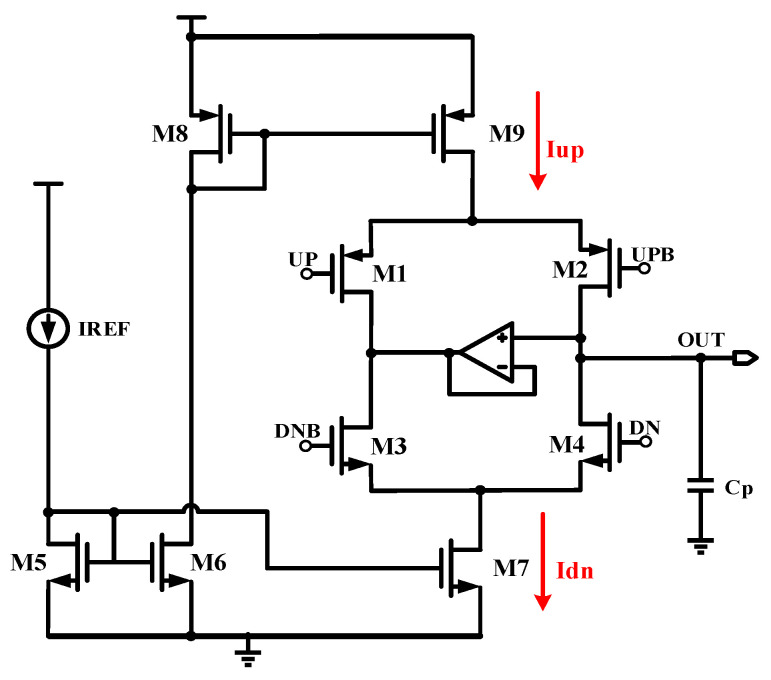
Traditional op-amp charge pump.

Figure 2 shows the circuit structure of a conventional op-amp charge pump. The differential switching signals UP, UPB, DNB, and DN control turning the MOS transistors M1, M2, M3, and M4, respectively, on and off, thereby determining the current path of the charge pump. When transistors M2 and M3 are turned on and transistors M1 and M4 are turned off, the charging branch of the charge pump is turned on. The charging current flows to the output through transistor M2, increasing the output voltage. At the same time, the current flowing through M3 will not affect the output of the charge pump. When transistors M1 and M4 are turned on and M2 and M3 are turned off, the charge pump begins discharging through transistor M4. Finally, when M2 and M4 are both in the off state, neither the charging nor discharging branches exist, and the output voltage remains at a fixed potential. The unity gain negative feedback amplifier in the figure acts as a clamp to stabilize the output voltage. However, some non-ideal effects in this charge pump circuit can cause high ripple in the output voltage even when the loop is locked.

1. Switching time mismatch

This effect is induced by the charge pump’s distinct types of switch tubes in the charging and discharging branches. As illustrated in Figure 2, the charging branch’s switching transistor is a PMOS, while the discharging branch’s switching transistor is an NMOS. Different types of switching transistors have inconsistencies in their turn-on and turn-off periods, and then the asynchronous control signal can cause the succeeding VCO to generate aperiodic waveforms [16].

2. Current mismatch

Current mismatch occurs when the charge pump’s charging current Iup and discharging current Idn are not precisely equal; even if the pulses of the control signal UP and DN are properly synchronized, the net current created by the charge pump is not zero, which causes the output voltage OUT to increase by a fixed value at each phase comparison moment. However, the average output voltage of the charge pump needs to stay constant for the PLL to stay locked. As a result, the phase-locked loop generates a phase error between the input and output, which will cause periodic ripples in the output voltage of the charge pump. In short, the mismatch of the charge and discharge currents caused by MOS devices can be obtained as follows:

(2) $$\frac{\Delta I_{\mathrm{cp}}}{I_{\mathrm{cp}}}=\left|\frac{\Delta I_{\mathrm{up}}}{I_{\mathrm{up}}}-\frac{\Delta I_{\mathrm{dn}}}{I_{\mathrm{dn}}}\right|$$

where Δ*I*_up_ is the charging current mismatch, and Δ*I*_dn_ is the discharging current mismatch.

3. Clock feedthrough

**Figure 3:**
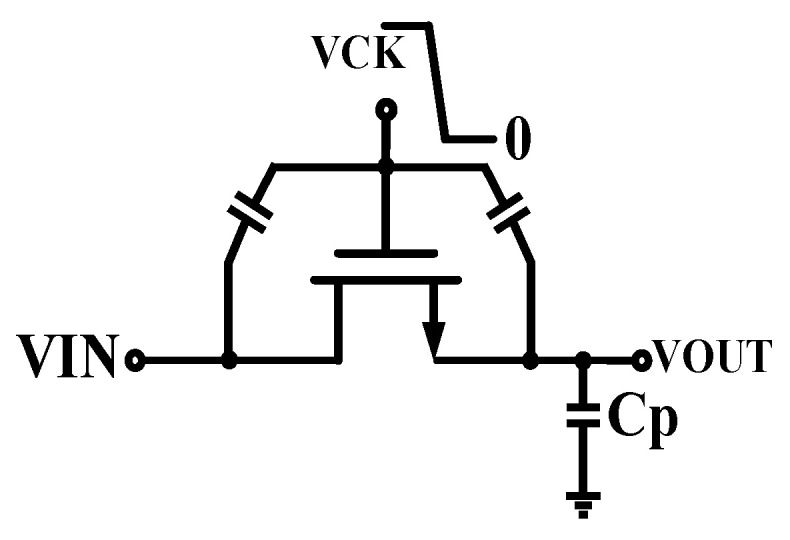
Diagram of clock feedthrough effect.

As shown in Figure 3, the switch tube has a gate-to-drain parasitic capacitance C_gd_. The clock signal VCK is coupled to the loop filter through the parasitic capacitance, which, in turn, interferes with the VCO control voltage and causes ripples in the output voltage VOUT [17].

4. Charge Injection

**Figure 4:**
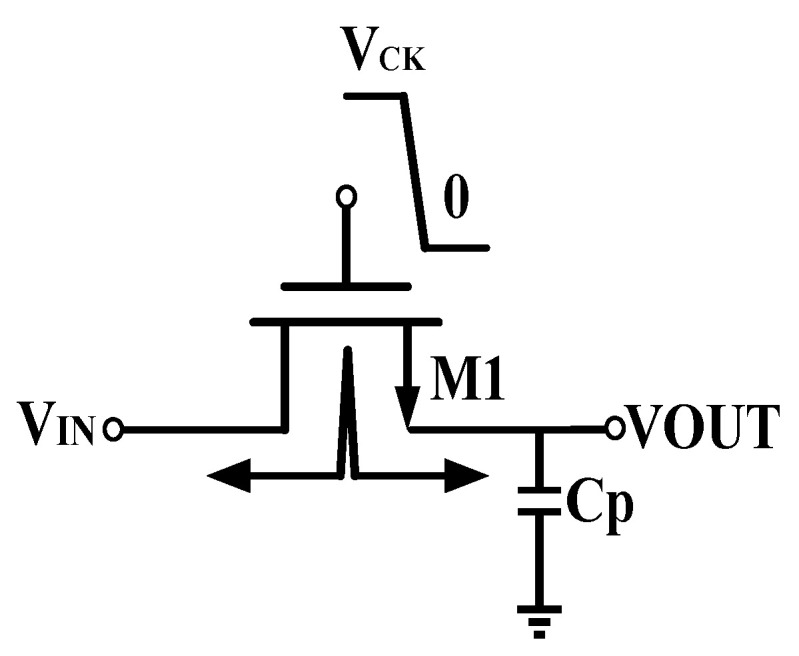
Diagram of channel charge injection effect.

Figure 4 is a schematic diagram of the channel charge injection effect. When the MOS transistor M1 is turned on, its source and drain voltages are approximately equal, that is, *V*_IN_ ≈ *VOUT*. The total charge in the inversion channel *Q*_ch_ [18] is as shown in Equation (3):

(3) $$Q_{\mathrm{ch}}=WLC_{\mathrm{ox}}(V_{\mathrm{CK}}-V_{\mathrm{IN}}-V_{\mathrm{TH}})$$

In the circuit depicted in Figure 4, when transistor M1 is off, the charge *Q*_ch_ may leak through the source and drain off the transistor. This process is called channel charge injection. When the charge is injected into the drain, it is usually absorbed by the input without significantly impacting the circuit’s normal operation. However, if the charge injection occurs at the source of M1, these charges will accumulate at the capacitor *C*p. As the charge accumulates, a voltage error will occur in the output voltage VOUT. Assuming that all the charges are injected into the capacitor *C*p, the error voltage generated is

(4) $$\Delta V=\frac{WLC_{\mathrm{ox}}(V_{\mathrm{CK}}-V_{\mathrm{IN}}-V_{\mathrm{TH}})}{Cp}$$

### 2.2. A Conventional Source-Switching Charge Pump

**Figure 5:**
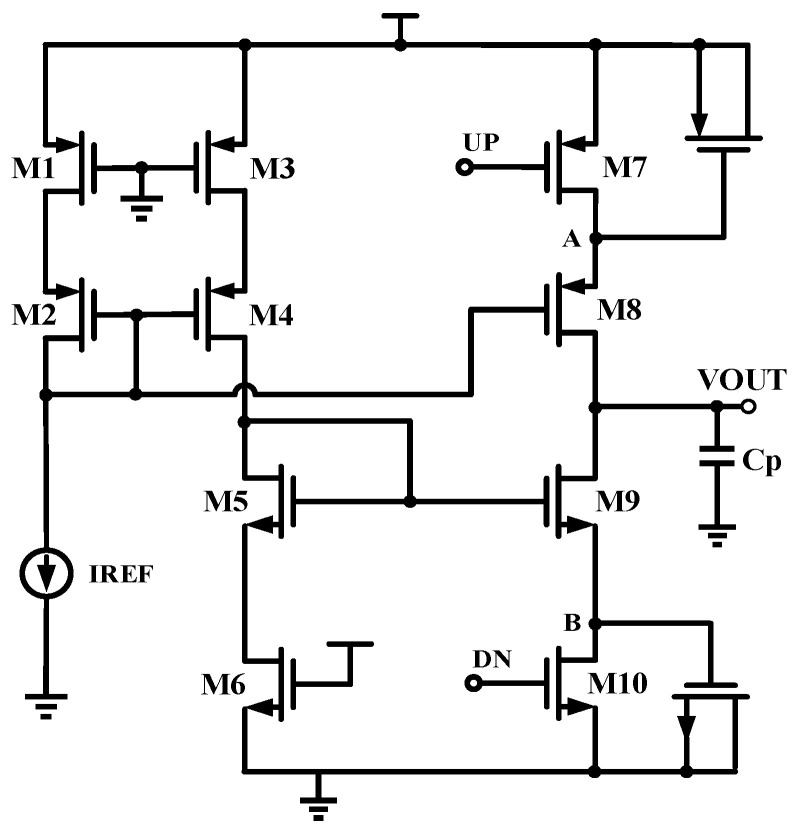
Conventional source-switching charge pump.

Figure 5 is a conventional source-switching charge pump. M7 and M10 are switching transistors, and their on/off status are controlled by the switching signals DN and UP. When M7 is turned on, the current IREF is mirrored to M8 through M2, charging the capacitor *C*p. Similarly, when M10 is turned on, the current IREF is mirrored to M9 through M5 to act as the discharge current, releasing the charge at the capacitor *C*p. Therefore, the charge and discharge of the circuit can achieve a good match due to the equivalent current values of M5 and M4. However, this circuit also has some non-ideal effects. For example, the frequent switching of the switching transistors induces the effects of charge injection and charge sharing, resulting in significant fluctuations in the voltages of nodes A and B. In addition, when the output voltage rises to a potential close to VDD, M8 will enter the linear region; and when the output voltage drops to a potential close to VSS, M9 will enter the linear region. However, there will be a significant current mismatch if transistors M8 or M9 enter the linear zone, which will cause the charge pump output to fluctuate in the locked state. Generally, long-channel transistors are preferred to minimize this effect. However, extending the length of the transistors will result in higher parasitic capacitances and a slower charge pump response.

## 3. Proposed Charge Pump Circuits

**Figure 6:**
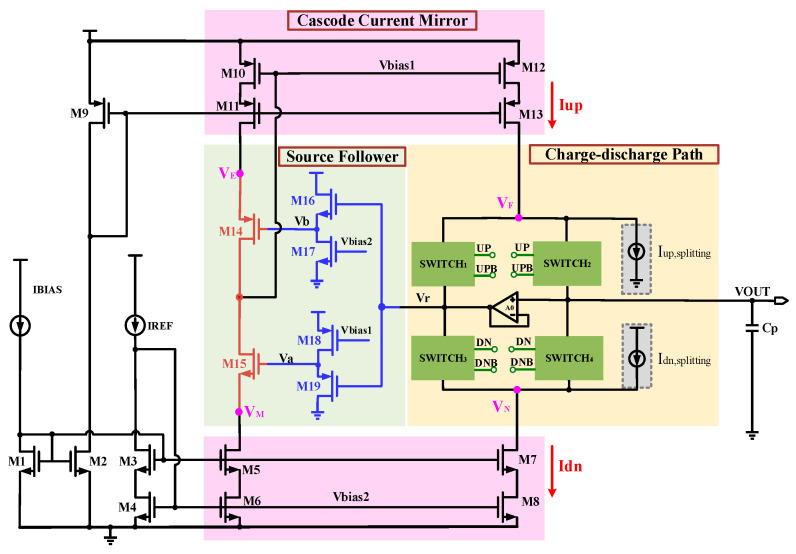
Proposed charge pump circuit.

Through the analysis of the aforementioned traditional charge pump circuits, it is evident that there are numerous non-ideal effects in traditional charge pumps, including current mismatch, clock feedthrough, charge sharing, and charge injection, all of which contribute to undesirable outputs for charge pumps. Based on this, a novel low-current mismatch charge pump circuit is proposed in this paper, as shown in Figure 6. This circuit features a differential input and a single-ended output configuration, primarily composed of a cascode current mirror, a charge–discharge path, and a source follower. A cascode current mirror with high output impedance is employed to mitigate the impact of MOS channel length modulation effects, enhancing the current accuracy of the charge pump. Four transmission gates are utilized in the charge–discharge path to control the replenishment and discharge of charge in the output capacitor. A source follower structure is introduced to minimize the current mismatch. Also, a low threshold voltage transistor is used to build the charge pump to ensure the output margin of this circuit.

### 3.1. T-Shaped Analog Switches

**Figure 7:**
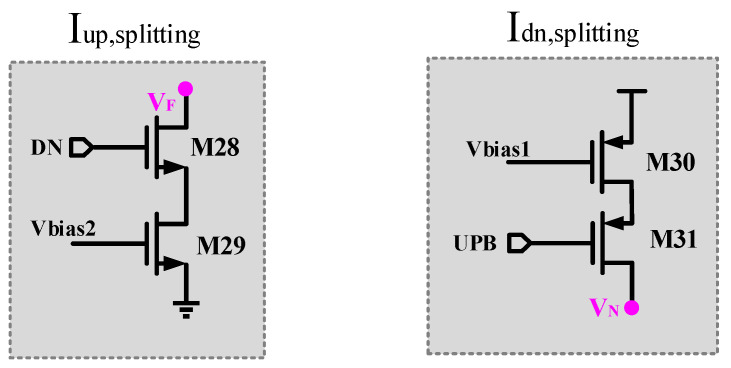
Current-splitting circuit.

The new structure proposed in this paper uses T-shaped analog switches to act as transmission gates, which can suppress the non-ideal effects of clock feedthrough, switch timing mismatch, and charge injection. This paper draws on the technology for suppressing current mismatch proposed in the literature [19], adding two current-splitting circuits, which are marked in gray, and they are controlled by the signals DN and UPB, as shown in Figure 7. When the DN signal is at a high level and the UPB signal is low, the controlled branches where M28 and M31 are located will divert a portion of the current from Iup and Idn, respectively, to reduce the total current applied to the output, thereby achieving the purpose of reducing the current imbalance. In addition, in the static operating state of the circuit, the current-splitting circuits will be closed to maintain a larger output static current.

**Figure 8:**
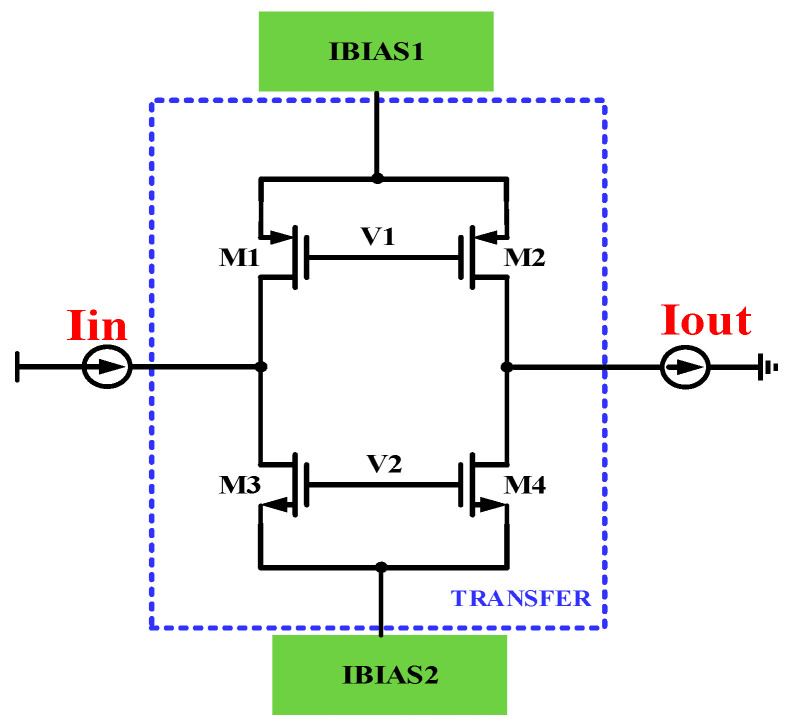
T-shaped analog switches.

The T-shaped analog switches SWITCH_1_ and SWITCH_2_ are controlled by the signals UP and UPB, and SWITCH_3_ and SWITCH_4_ are controlled by the signals DN and DNB. The structure of the T-shaped analog switches is shown in Figure 8. Modules IBIAS1 and IBIAS2 provide the current bias for the transmission gates. The PMOS transistors M1/M2 and the NMOS transistors M3/M4 form the current transmission gate. When V1 is low and V2 is high, the transmission gate is conductive, and the input current Iin flows through the transmission gate. The channel of the NMOS transistor consists of negatively charged electrons, while the channel of the PMOS transistor consists of positively charged holes. When the transmission gate is in a closed state, these electrons and holes undergo compounding. This compounding process reduces the net charge entering the following loop filter. Furthermore, when the transmission gate closes and opens, the voltages across the PMOS and the NMOS are in opposite directions, thus reducing the clock feedthrough effect. Since each transmission gate consists of both the NMOS and the PMOS, it effectively solves the problem of switching time mismatch caused by the different types of switch tubes in the charging and discharging branches.

In addition, the problem of charge injection has been perfectly solved. Each pair of NMOSs and PMOSs in the T-shaped analog switches has complementary control signals. Therefore, even if the ratio of the PMOS and the NMOS is not specially set, when the control signals of the switches are flipped, the charge that should be injected into one transistor is absorbed by the other transistor, thus eliminating the error voltage at the output due to charge injection. When the UP and DN signals are low, SWITCH_2_ and SWITCH_4_ are off, SWITCH_1_ and SWITCH_3_ are on, the capacitor Cp is not charged or discharged, and the output voltage remains constant. The charge pump current flows only through SWITCH_1_ and SWITCH_3_, ensuring that the current source can operate continuously when the charge pump is in the hold state. As a result, the current source can quickly charge and discharge the output capacitor when SWITCH_2_ and SWITCH_4_ are turned on, reducing the time required to build up the charging and discharging currents when SWITCH_2_ and SWITCH_4_ are on.

**Figure 9:**
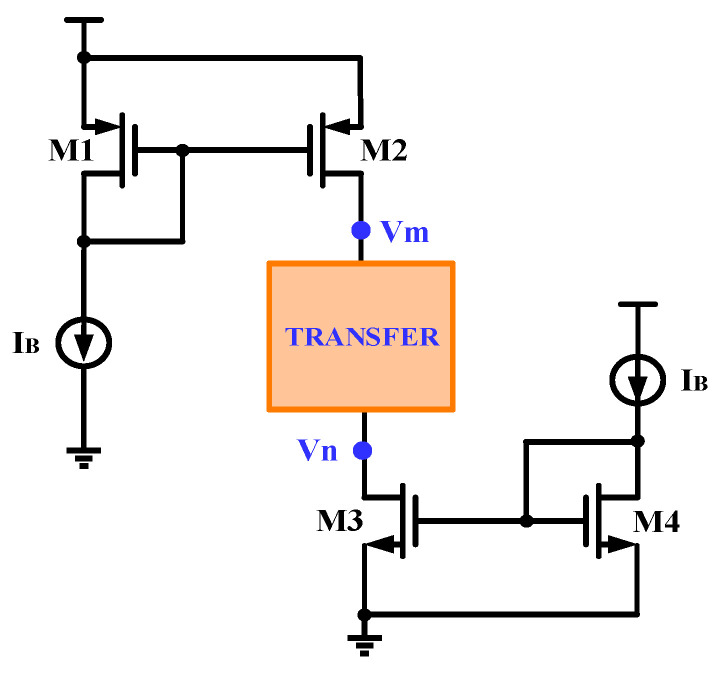
Structure of conventional current bias.

The modules IBIAS1 and IBIAS2 in Figure 8 provide current bias for the T-type analog switches so that there is definite potential at the source terminals of M1-M4, which helps the switches turn on and off quickly. The bias current provided by IBIAS1 needs to be equal to the current from IBIAS2, and only in this way can the Iin current be accurately transferred to Iout. Figure 9 is the structure of traditional current bias; due to the channel length modulation effect, when the voltages of Vm and Vn are high, the current mirrored by the M2 transistor may be smaller than the current mirrored by the M3 transistor; when the voltages of Vm and Vn are low, the current mirrored by M2 will be larger than the current mirrored by M3.

**Figure 10:**
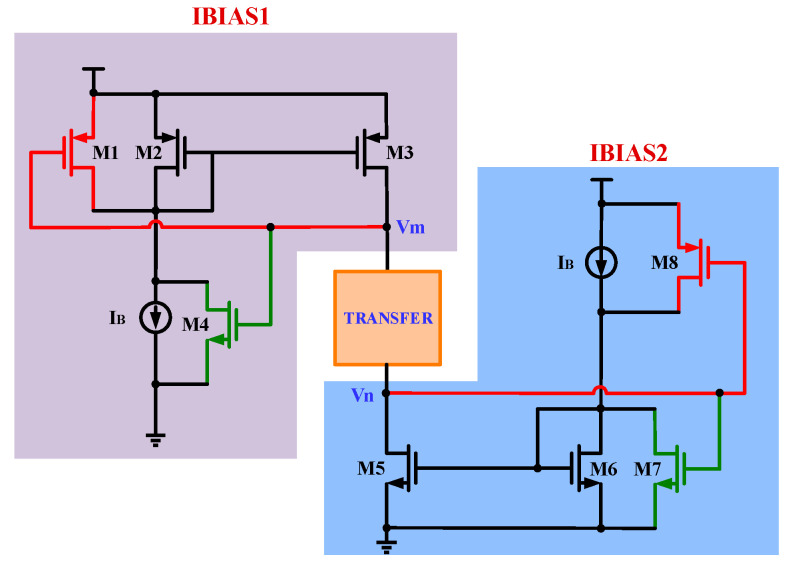
Structure of dual-feedback current mirror.

In order to improve the matching accuracy of the bias current, this paper proposes a structure of dual-feedback current mirrors, as shown in Figure 10. For the IBIAS1 module, M2 and M3 constitute the current mirror, and M1 and M4 are two negative feedback MOS tubes. If the voltage of Vm is too low, the Vds of M3 become very large; at this time, the feedback tube M1 splits part of the current, and then the gate-source voltage of M2 decreases, which reduces the Vgs of M3. Thus, the current replicated by the current mirror can be reduced; this further alleviates the increase in the charging current caused by the long channel effect to a certain extent and suppresses the current deviation. When the voltage of Vm is too high, the Vds of M3 drops, and if Vds is lower than the overdrive voltage, the current mirror tube M3 will enter the linear region, causing the charging current to decrease sharply. In this paper, the feedback tube M4 is utilized to generate a compensation current when the voltage of Vm is very high, increasing the current to M2, which is equivalent to decreasing the drain voltage of M2, and the gate-source voltage of M3 will rise, thus compensating for the diminution in the charging current. Similarly, for the IBIAS2 module, when the voltage of Vn is too low, the feedback tube M8 acts to increase the current replicated by M5; when the voltage of Vn is too high, the feedback tube M7 can be used to lower the discharge current of M5 by splitting away part of the current. Thus, the proposed dual-feedback current mirror structure is able to provide accurate current biasing for T-type analog switches.

### 3.2. Source Follower

**Figure 11:**
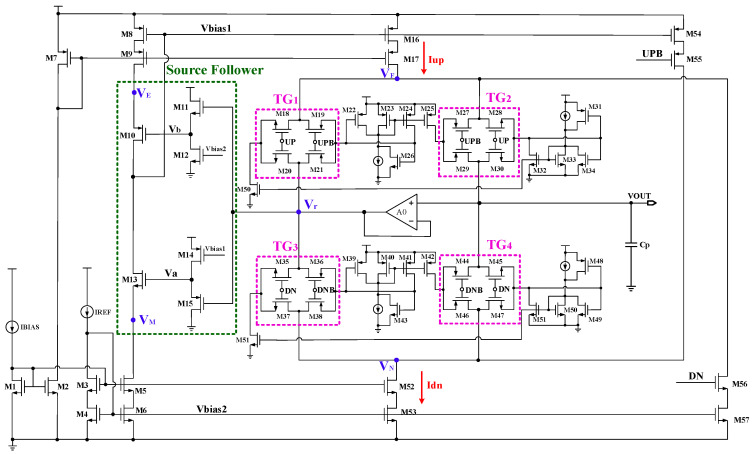
Schematic diagram of the novel charge pump.

The input voltage of a source follower is in phase with the output voltage, and the voltage gain is approximately 1. Therefore, the proposed charge pump circuit utilizes a source follower architecture to track the voltage, ultimately ensuring high current matching. Figure 11 shows a schematic of the novel charge pump, where the charging current Iup and discharging current Idn are generated by mirroring the current IREF. The switching tube operates in the linear region when it conducts, and its drain-source voltage is related to the current as follows: *V*_ds_ = *I*_ds_/*g*_ds_. The *V*_ds_ of the switching tube is also kept constant with a constant reference current IREF. There is the following relationship between nodes *V*_F_, *V*_N_, and *V*_r_:

(5) $$V_{F}=V_{r}-\left|V_{\mathrm{ds},M30}\right|+\left|V_{\mathrm{ds},M28}\right|$$

(6) $$V_{N}=V_{r}-\left|V_{\mathrm{ds},M45}\right|+\left|V_{\mathrm{ds},M47}\right|$$

The charge pump proposed in this paper enhances the current matching by utilizing a source follower consisting of M11/M12 and M14/M15. Transistor M15 enables the voltage of Va to follow the change in *V*_r_, and then the voltage of Va is used as the gate control signal for M13 so that the voltage of *V*_M_ can finally follow the change in *V*_r_. The relationship between *V*_M_ and *V*_r_ can be obtained:

(7) $$V_{M}=V_{r}+\left|V_{\mathrm{gs},M15}\right|-V_{\mathrm{gs},M13}$$

Similarly, the voltage *V*b can follow the variation in *V*_r_ through M11 and then control the gate of M10, ultimately making V_E_ follow the change in *V*_r_. The expression for the relationship between *V*_E_ and *V*_r_ is as follows:

(8) $$V_{E}=V_{r}-V_{\mathrm{gs},M11}+\left|V_{\mathrm{gs},M10}\right|$$

By adjusting the width and length of M13 and M15, *V*_M_ *= V*_N_ can be obtained; similarly, *V*_E_ *= V*_F_ can be realized by appropriately setting the size of M10 and M11. Therefore, the charging and discharging currents can be well matched in the new charge pump. At different output voltages, the charging current Iup and the discharging current Idn can be precisely equal to the current IREF. The voltage range of *V*_r_ can be obtained as follows:

(9) $$V_{\mathrm{dsat},M11}+V_{\mathrm{th},M12}<V_{r}<VDD-\left|V_{\mathrm{dsat},M14}\right|-\left|V_{\mathrm{th},M15}\right|$$

With a high gain amplifier A0, *V*_r_ *= VOUT*, so the output voltage range of the charge pump is

(10) $$V_{\mathrm{dsat},M11}+V_{\mathrm{th},M12}<VOUT<VDD-\left|V_{\mathrm{dsat},M14}\right|-\left|V_{\mathrm{th},M15}\right|$$

From the above equation, it can be seen that the range of the output voltage is limited by the threshold voltages of M12/M15 and the overdrive voltages of M11/M14. Therefore, MOS transistors with low threshold voltages should be selected for M12 and M15, and the overdrive voltages of M11 and M14 should be as small as possible. The current error in this circuit can be expressed as follows:

(11) $$\Delta I_{\mathrm{dn}}=\lambda_{n}(V_{N}-V_{M})\cdot \mathrm{IREF}$$

(12) $$\Delta I_{\mathrm{up}}=\lambda_{p}(V_{F}-V_{E})\cdot \mathrm{IREF}$$

Therefore, the current mismatch ratio between I_up_ and I_dn_ is

(13) $$\sigma =\left|\frac{\Delta I_{\mathrm{dn}}-\Delta I_{\mathrm{up}}}{\mathrm{IREF}}\right|=\left|\lambda_{n}(V_{N}-V_{M})-\lambda_{p}(V_{F}-V_{E})\right|\times 100\%$$

According to the above analysis, the charge pump can achieve minimal current mismatch in the entire output voltage range by using the two-stage source follower to make *V*_N_ ≈ *V*_M_ and *V*_E_ ≈ *V*_F_. 

### 3.3. The High-Gain Rail-to-Rail Input Amplifier

The primary function of the unity gain amplifier A0 in Figure 11 is to eliminate the charge-sharing effect of the charge pump. Since the gain amplifier forms a unity gain feedback loop, it ensures that the voltage between nodes V_r_ and VOUT always remains equal. When both transmission gates TG_2_ and TG_4_ are turned off, the system reaches a locked state, and then the voltages of nodes V_F_ and V_N_ are equal, both close to the voltage of VOUT. Therefore, when the transmission gates TG_2_ and TG_4_ are turned on at the same time, the voltages of V_F_ and V_N_ will not change much, so the nodes V_F_ and V_N_ do not obtain charges from the parasitic capacitance. This shows that when the transmission gate is in operation, the loop filter does not contribute any additional charge to the circuit and therefore can effectively eliminate the charge-sharing effect.

**Figure 12:**
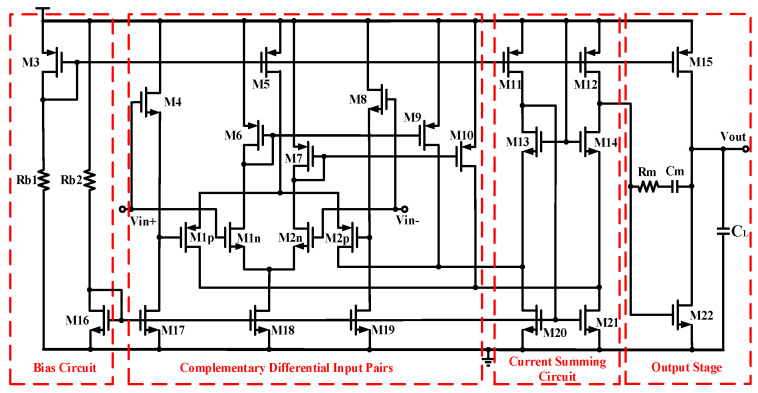
Schematic diagram of high-gain rail-to-rail input amplifier.

In order to achieve an accurate match between the charging and discharging currents, amplifier A0 should have a high gain because the current mismatch is determined by the size and gain of the core circuit [20]. Additionally, the output of the charge pump in this design will fluctuate over a wide range; thus, the input to the amplifier needs to be able to achieve the rail-to-rail input common-mode voltage. As shown in Figure 12, M1n and M2n are NMOS input transistors, and M1p and M2p are PMOS input transistors. These two pairs of input transistors form a rail-to-rail input structure, which can meet the input voltage range of 0~VDD. The gain A_V_ and main pole *f*_pd_ of the amplifier can be obtained as follows:

(14) $$A_{V}=(g_{m1p}+g_{m1n})r_{\mathrm{ds}12}g_{m22}(r_{\mathrm{ds}22}//r_{\mathrm{ds}15})$$

(15) $$f_{pd}=\frac{1}{2\pi g_{m22}(r_{\mathrm{ds}22}//r_{\mathrm{ds}15})C_{m}r_{\mathrm{ds}12}}$$

## 4. Simulation Results

**Figure 13:**
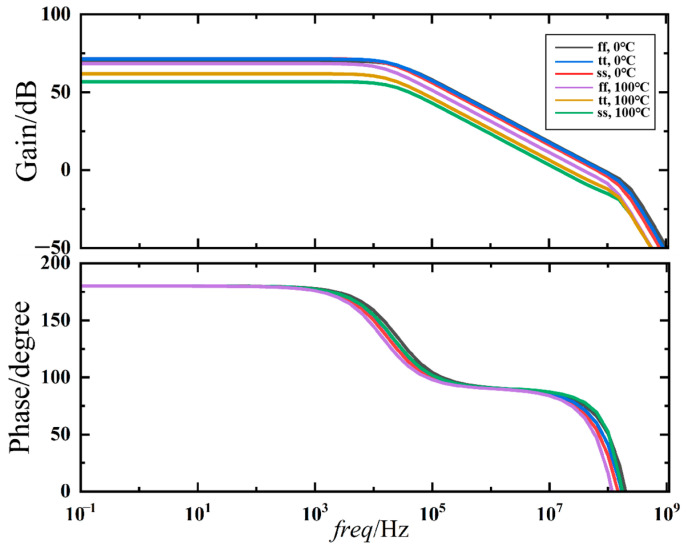
Stability simulation results for high-gain rail-to-rail input amplifier.

The proposed charge pump circuit is simulated using the 65 nm BCD process and the Cadence Virtuoso IC618. The high-gain error amplifier is designed to ensure the good performance of the charge pump, mitigating current mismatch and current variation across different temperatures, processes, and supply corners. Stability analysis has been conducted as the temperature varies from 0 °C to 100 °C under three process corners, ss (Worst Case), tt (Typical Case), and ff (Best Case). The results are shown in Figure 13; the phase margin varies from 53 to 83 degrees, and the loop gain ranges from 57 dB to 71 dB. At extreme process corners, the op-amp will operate at an inappropriate operating point, which can change the output impedance and equivalent Gm. Nevertheless, in most cases, the loop gain is above 70 dB.

**Figure 14:**
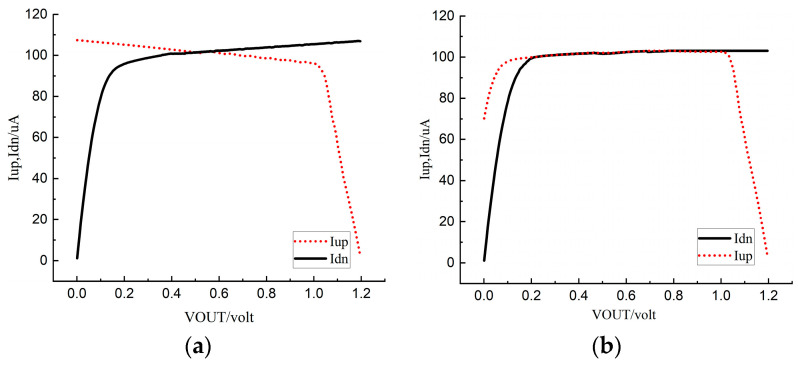
Simulation results for current mismatch: (**a**) traditional charge pump; (**b**) novel charge pump.

Figure 14 simulates the current mismatch between the traditional and new charge pump circuits under a 100 μA reference current. Figure 14a shows the charging current Iup and the discharging current Idn of the traditional charge pump circuit as the output voltage VOUT changes. As can be seen from the figure, the charging current and the discharging current of the traditional charge pump circuit are quite different. When VOUT changes from 0.2 V to 1 V, the variation range of Iup and Idn is from 95.7 μA to 105.3 μA, with a change of 9.6 μA, and the maximum current mismatch rate reaches 9.2%. Figure 14b shows the current variation of the new charge pump under typical condition. It can be seen from the figure that when the output voltage VOUT is between 0.2 V and 1 V, the charging current and the discharging current of the new charge pump structure match well. The variation range of Iup and Idn is 99.8 μA to 102.3 μA, with a change of 2.5 μA, and the current mismatch rate is only 0.21%.

**Figure 15:**
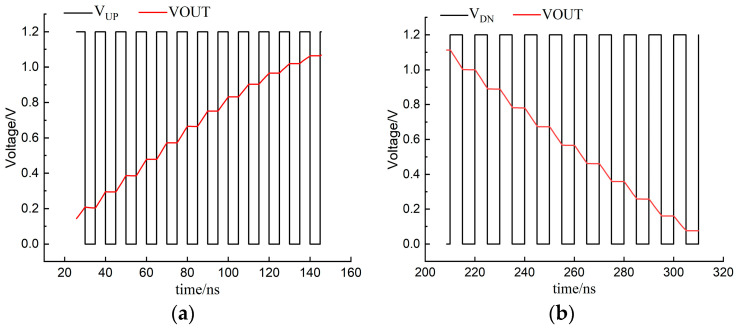
Waveform diagram of charge pump input signal and output voltage VOUT: (**a**) V_UP_ and VOUT; (**b**) V_DN_ and VOUT.

**Figure 16:**
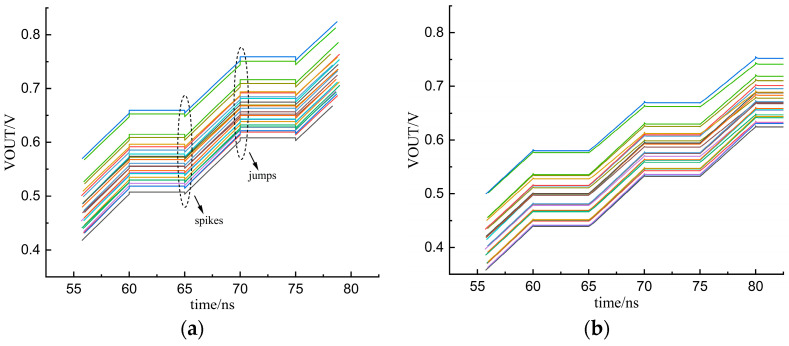
Output voltage VOUT for PVT conditions: (**a**) the traditional charge pump; (**b**) the novel charge pump.

Figure 15 shows the input signal UP/DN and the output voltage waveform of the charge pump. As can be seen from Figure 15a, when the UP signal becomes high, the output voltage increases; when the UP signal is low, the output voltage remains unchanged. In Figure 15b, VOUT decreases when V_DN_ is high, and VOUT remains constant when V_DN_ is low. In order to demonstrate more graphically the improvement in the clock feedthrough effect using the new charge pump circuits, Figure 16 shows the output voltages of the traditional op-amp charge pump and the new charge pump under PVT conditions (process angles ss, ff, and tt, temperature variations of 0 °C to 100 °C, and supply voltage fluctuations of 10%). Figure 16a is the output voltage waveform of the traditional op-amp charge pump. It shows that when the UP signal changes, the output voltage obviously jumps under five different process angles. Figure 16b indicates that when the UP signal changes, the output voltage of the new charge pump circuit does not have spikes and jumps. Therefore, it can be concluded that the new charge pump circuit significantly improves the clock feedthrough effect.

**Figure 17:**
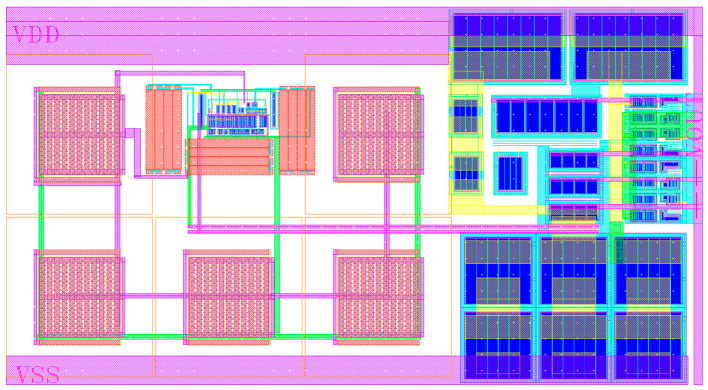
Layout of the novel charge pump.

The layout of the new charge pump is shown in Figure 17, with an area of 217 × 117 μm^2^. The performance parameters of the charge pumps in this paper and other works are compared in Table 1. It can be found from Table 1 that at an operating current of 100 μA, the current mismatch ratio and the current variation of the charge pump in this paper are only 0.21% and 1.4%, respectively. It has a lower current mismatch than charge pumps in references [11,21,22,23]. Although the charge pump in reference [24] has a low current mismatch, its current variation is significant over the output range.

## 5. Conclusions

This paper presents a charge pump circuit with a wide output range and low current mismatch applied to phase-locked loops. Based on the technical defects in traditional CPs, the transmission gate structure of T-shaped analog switches is adopted to enhance the immunity of the charge pump circuit to non-ideal effects such as clock feedthrough and charge injection. A source follower and current splitting circuits are proposed, which is combined with an amplifier structure of high gain rail-to-rail input to significantly improve the matching of the charge and discharge currents. In the 65 nm CMOS process, with a power supply voltage of 1.2 V and a current source of 100 μA, the maximum current mismatch rate and the current variation rate of the new charge pump are only 0.21% and 1.4%, respectively. And the output voltage range of the proposed charge pump is 0.2~1 V. Compared with other charge pump circuits reported in the existing literature, the design proposed in this paper not only provides a wider voltage output range but also achieves a lower current mismatch rate. The shortcoming of this paper is that it has not been demonstrated whether the proposed charge pump can function appropriately in PLL applications. Analysis and verification of this will be undertaken in future work.

## Figures and Tables

**Table 1:** Comparison of performance of proposed charge pump with previous works.

Parameter	This Work	[11]	[21]	[22]	[23]	[24]
Year	2024	2021	2018	2016	2023	2008
Tech. (nm)	65	45	180	180	65	180
Supply (V)	1.2	1.8	1.8	1.8	1.2	1.8
Icp (μA)	100	10	10	100	80	200
Compliance range (V)	0.2–1	0.13–1.63 V	0.25–1.25	0.3–1.5	0.24–0.9	0.25–1.62
I-Mismatch (%)	0.21	<2	0.57	0.32	1.75	0.0105
I-Variation (%)	1.4	1.2	1	<3.5	NA	16.8

## Data Availability

The original contributions presented in the study are included in the article, further inquiries can be directed to the corresponding author.
